# Hydrocarbons removal from synthetic bilge water by adsorption onto biochars of dead *Posidonia oceanica*

**DOI:** 10.1007/s11356-022-21998-x

**Published:** 2022-07-22

**Authors:** Salvatore Cataldo, Nicola Muratore, Francesco Giannici, David Bongiorno, Vitaliano Chiodo, Susanna Maisano, Alberto Pettignano

**Affiliations:** 1 Dipartimento di Fisica e Chimica – Emilio Segrè, Università di Palermo, V.le delle Scienze, ed. 17, 90128 Palermo, Italy; 2 Dipartimento di Scienze e Tecnologie Biologiche, Chimiche e Farmaceutiche (STEBICEF), Università di Palermo, V.le delle Scienze, ed. 17, 90128 Palermo, Italy; 3 Istituto CNR-ITAE, via Salita S. Lucia sopra Contesse 5, 98126 Messina, Italy

**Keywords:** Hydrocarbon, Biochar, *Posidonia oceanica*, Adsorption, Bilge water

## Abstract

Bilge waters are wastewaters produced on boats during navigation and usually contain hydrocarbons and oils. They cannot be directly released into the sea if not below a hydrocarbons concentration limit set by current legislation. Appropriate oil in water separator (OWS) systems can be installed on board boats to remove hydrocarbons from bilge water allowing their spillage into the sea. These systems may contain an adsorption step on a suitable adsorbent. Here, biochars produced from pyrolysis of dead *Posidonia oceanica*, pristine or chemically activated, have been tested as hydrocarbons adsorbents. Adsorption experiments with aqueous dispersions simulating bilge waters containing a marine gas oil (MGO) fuel for boats, a surfactant, and different NaCl concentrations were carrying out. The hydrocarbons concentrations before and after adsorption have been directly measured by using the reverse phase HPLC technique coupled with a fluorescence detector. These measurements are very fast and their reliability was verified by re-measuring the hydrocarbons concentrations of some samples with the GC–MS-MS technique, according to one of the traditional methods for hydrocarbons determination in emulsions. Different isotherm equations were used to fit the adsorption data. The biochars were characterized from the chemical-structural point of view by means of several instrumental techniques.

**Supplementary Information:**

The online version contains supplementary material available at 10.1007/s11356-022-21998-x.

## Introduction

Bilge waters are essentially emulsions of oils and hydrocarbons in water, fresh or salty, in the presence of substances with emulsifying properties and are the result of normal operations taking place on board boats during navigation (Karakulski et al. 1995; Tomaszewska et al. 2005). The sources of the various components of these wastewaters are many: propeller oils, emptying of containers for hydrocarbons, detergent, and degreasing substances. The current legislation, Marpol 73/78 (International Maritime Organization (IMO) 2011), provides for a maximum concentration limit of total hydrocarbons, 15 mg L^−1^, below which the bilge water can be spilled directly into the sea (MEPC 2003). The same legislation indicates the specifications for bilge water treatment systems to be loaded on ships. These systems (oil in water separators, OWS) have the purpose of treating bilge water so that the concentration of hydrocarbons is reduced below the limit allowed for spillage into the sea.

To monitor the total concentration of hydrocarbons (TPH) in continuous systems, different techniques including turbidity measurement and fluorescence detections are used. These detectors convert the measured signal into TPH using appropriate calibration functions and proper approximation. For example, in fluorescence detectors, the signal came from aromatic components of hydrocarbon mixtures, but it is considered attributable to a certain quantity of total hydrocarbons on the assumption that the ratio between fluorescent and non-fluorescent fractions remains constant during the purification treatments. Measurement systems such as oil in water analyzer (OIW) or oil content monitor (OCM) are a constituent part of the OWS and control the solenoid valve that determines the discharge into the sea or the return of the treaty upstream of the plant (MEPC 2003).

Physical processes are currently the most used methods of bilge water treatment in OWS and include the use of centrifuges, coalescing filters, and flotation systems (Albert and Danesi 2003). The oil in water separator must be able to perform the remediation even of a large quantity of bilge water depending on the tonnage of the ship always maintaining adequate compactness. Moreover, the irreversible dissolution of organic materials, as well as the presence of detergents can be limiting factors in the separation process (Cazoir et al. 2012). To solve this kind of problem, it is possible to add to the OWS a further purification step based on the filtration through suitable adsorbent materials like clays and/or activated carbons. This last purification step can also represent the unique bilge water treatment carried out on boats with a gross tonnage of fever than 400 tons. Furthermore, the introduction in OWS of an adsorption step can be crucial considering that the ever-greater environmental sensitivity pushes towards the development of increasingly efficient separation systems for obtaining post-treatment water with a hydrocarbon content lower than 5 mg L^−1^.

During the last decades, several natural and synthetic adsorbent materials for the removal of hydrocarbons from wastewater have been proposed in a large number of articles and reviews (Yousef et al. 2020; Zapata et al. 2020; Albatrni et al. 2019).

The main objective of this work was to test a biochar of dead *Posidonia oceanica* residues as adsorbent material of hydrocarbons from emulsions prepared with a surfactant and a marine gas oil (MGO) fuel for boats (DMA type, ISO 8217:2017 (ISO 2017)), that simulated the bilge water produced during ship navigation.

Biochars are vegetable carbons obtained from the pyrolysis of different types of biomass (Kwapinski et al. 2010; Maisano et al. 2017) and are considered a by-product of renewable fuel production (Yaashikaa et al. 2020). They are composed mainly of amorphous carbon whose surface is highly functionalized and reactive towards many organic and inorganic compounds (Qian et al. 2015). *Posidonia oceanica* is a Mediterranean sea plant (Chiodo et al. 2016). Considering that beached leaves of *Posidonia oceanica* are present in large quantities on the Sicilian coast and are available for free, the use of its pyrolysis by-product is even more economically advantageous.

The biochar of *Posidonia oceanica* was tested as it was (biochar pristine, BCP) and also after two types of chemical activation, i.e., an acid activation by impregnating the material with sulfuric acid (ABCP) and a basic activation with potassium hydroxide (BBCP) (Cataldo et al. 2018; Pedicini et al. 2020). As known, chemical activation processes enhance the adsorption ability of biochars (Zhang et al. 2014; Vithanage et al. 2015) and an intensification of the adsorption ability of BCP towards toxic metal ions was already observed for its activated form ABCP (Cataldo et al. 2018). In this work, a commercial activated carbon, the Filtrasorb 400, has been used for comparison purposes.

Several batch and column adsorption experiments have been carried out on emulsions containing the hydrocarbons and two different surfactants, i.e., sodium dodecyl sulfate (SLS) and sodium dodecylbenzenesulfonate (SDBS). The SLS is one of the most used surfactant in cleaning products, while SDBS is the surfactant used in the preparation of test fluids employed in the OWS testing, as required by the current legislation (MEPC 2003) (see SLS and SDBS molecular structures in Fig. SM1 of Supplementary Material).

The presence of surfactants increases the hydrocarbons dispersion hindering the separation processes in OWS. For this reason, the four adsorbent materials here used have been tested through adsorption experiments in the worst conditions, i.e., with surfactant concentrations higher than the critical micellar concentration (CMC), with the idea that the last hydrocarbons removal step with a lower surfactant content can only give better results than those here obtained.

Bilge waters often contain also a fairly variable percentage of seawater which increases its salt content. For this reason, the dependence on the salinity of the biochars adsorption capacity towards hydrocarbons was taken into account carrying out a series of batch experiments with emulsions containing also different amounts of NaCl.

Another aim of this work was to measure the hydrocarbons concentration accurately and quickly in synthetic bilge water samples before and after adsorption experiments. To this end, total organic carbon (TOC) and HPLC with fluorescence detector (HPLC-FLD) techniques were chosen. Considering that HPLC-FLD reveals only that part of hydrocarbons that fluoresces, the validity of the results obtained was confirmed by re-measuring the concentration of hydrocarbons in some samples using the GC–MS–MS technique on the hydrocarbon fraction extracted with hexane, accordingly to the reference EPA method (US EPA 1999).

The biochars have been widely characterized from the chemical-structural point of view by means of elemental analysis, nitrogen adsorption–desorption analysis, SEM–EDX microanalysis, water contact angle measurements, FT-IR, XRD, and TGA techniques to obtain information useful in the understanding of adsorption mechanism.

The results obtained show that (i) the HPLC-FLD technique is suitable to rapidly and accurately measure the hydrocarbons concentration of a great number of water dispersions samples; (ii) the chemical activations treatments enhance the adsorption ability of biochar, especially the activation with KOH; (iii) the addition of a surfactant as well as the presence of salts to dispersions affect the removal capacity of the adsorbent materials; (iv) the high adsorption ability of BBCP makes this adsorbent material suitable for adsorption step of an OWS, even with bilge waters with a high surfactant content.

## Experimental section

### Chemicals

Synthetic bilge water was prepared by dispersing hydrocarbons in water with a surfactant. A marine gas oil (MGO) fuel for boats (DMA type) was used as a prototype of hydrocarbon mixture (Exon Mobil 2020). The surfactants alternatively used in the emulsion preparation were sodium dodecyl sulfate (SLS), (Sigma, ≥ 98%), and sodium dodecylbenzenesulfonate (SDBS), (Aldrich, technical grade). The salinity of emulsions was adjusted by adding different amounts of NaCl (Riedel-de Haën, puriss.). All the solutions/emulsions were prepared using freshly, CO_2_-free ultra-pure water (*ρ* ≥ 18 MΩ cm^−1^) and grade A glassware. The commercial activated carbon, Filtrasorb 400 (FS400) used for comparison purposes was supplied by Calgon-carbon. Acetonitrile (HiPerSolv CHROMANORM® for HPLC—SUPER GRADIENT Reag. Ph. Eur., USP, ACS water < 30 ppm—suitable for UPLC/UHPLC), water (HiPerSolv CHROMANORM® for HPLC), and formic acid (Fluka, eluent additive for LC–MS) were used in HPLC measurements. Perdeuterated eicosane (Cambridge Isotope Laboratories inc. atomic purity 98%), KCl (Sigma-Aldrich 99%), CaCl_2_ anhydrous (Supelco 98%), KOH (Sigma-Aldrich > 85,0%), hexane (VWR > 95%, Pestinorm), and anhydrous granular sodium sulfate (Merck EMSURE > 99%) were used in extraction-based GC–MS method.

### Preparation and characterization of biochars

Beached leaves of *Posidonia oceanica* collected from the southwestern coast of Sicily region (Italy) were properly washed, dried, and then carbonized through a stainless-steel fixed-bed reactor, at 400 °C for 1 h with a heating rate of 10 °C min^−1^ in a nitrogen atmosphere (150 mL min^−1^) by converting the sea plant to biochar (BCP) and bio-oil (Kwapinski et al. 2010; Maisano et al. 2017). The BCP can be considered a by-product of pyrolysis and was used as it was and also after two different chemical activation procedures, i.e., an acidic activation with H_2_SO_4_ (ABCP) and a basic activation with KOH (BBCP).

The two activation procedures are described in detail in refs (Cataldo et al. 2018) and (Maisano et al. 2017) and had the purpose of improving the performances of the biochar in terms of adsorption capacity towards hydrocarbons.

The three biochars together with the commercial activated carbon Filtrasorb 400 (FS400) have been extensively characterized.

The morphology was examined employing scanning electron microscopy (SEM) using a FEI XL30 microscope equipped with a field emission gun and EDX probe, operating at an accelerating voltage of 20 kV. The Brunauer–Emmett–Teller (BET) theory was used for the surface area determination where coverage of nitrogen molecules is assumed to be complete. Moreover, the pore size distribution was estimated by using the Barret-Joyner-Halenda (BJH) and Horvath-Kawazoe (HK) theories. Both surface area and average pore size measurements were carried out by analysis of N_2_ adsorption–desorption isotherms at -195.8 °C using a Micromeritics ASAP 2020 instrument. The preliminary outgassing treatment was performed under a vacuum (5 mmHg) at 90 °C for 1 h and then at 350 °C for 4 h under a high vacuum (100 mmHg).

Elemental analysis was performed using a Thermo Fisher Scientific (model Flash EA 1112) analyzer. In each measure, approximately 2 mg of sample was examined in terms of carbon, hydrogen, nitrogen, sulfur, and oxygen (CHNS-O) content.

Infrared absorption spectra were recorded in the 400 to 4000 cm^−1^ range with a Perkin-Elmer Frontier FT-IR spectrometer. The powders were dispersed in KBr and pressed into 13 mm pellets using 10 ton pressure. Thermogravimetric curves from 30 to 700 °C (5 °C min^−1^) were acquired using a TA Q5000 thermogravimetric analyzer (TA Instruments) under *θ*a nitrogen flow on a platinum sample holder. X-ray diffraction was acquired in Bragg–Brentano geometry with a Panalytical X’Pert Pro diffractometer using Ni-filtered Cu K-alpha radiation.

An optical contact angle apparatus (OCA 20, Data Physics Instruments) equipped with a video measuring system with a high-resolution CCD camera and a high-performance digitizing adapter was used to measure the water contact angle onto pellets of BBCP as it was and after contact for 24 h with aqueous solutions containing SLS (*c*_SLS_ = 6 g L^−1^) or SDBS (*c*_SDBS_ = 2.5 g L^−1^). SCA 20 software (Data Physics Instruments) was used for data acquisition. The contact angle was measured by the sessile drop method by gently placing a droplet of 12.0 ± 0.5 µL onto the surface of the pellets prepared with 100 mg of each powder sample previously dried in oven at 65 °C for 2 days and pressed at 10 tons for ca. 10 min. To minimize the effects of drop absorption on the material, the first detectable contact angle after oscillation of the droplet has stopped (*θ*_*i*_) was measured. A minimum of 3 droplets were examined for each surface.

### Hydrocarbons and hydrocarbons-surfactant quantification methods

Total organic carbon (TOC) measurements have been preliminarily used to measure the hydrocarbons and the hydrocarbons and surfactant concentrations in the emulsions.

A Shimadzu TOC-5050 system based on the combustion catalytic oxidation method coupled with a Shimadzu ASI-5000A autosampler was used for the TOC measurements. TOC values were attributed to the organic carbon coming from the sum of DMA and surfactant in the dispersions.

The poor reliability and the low selectivity of results obtained with this instrumental technique together with the usefulness of an analytical method capable of rapidly measuring the TPH in the dispersions have led us to quantify the hydrocarbons in dispersions using the reverse phase HPLC technique coupled with a fluorescence detector (HPLC-FLD).

The HPLC was an Agilent model 1260 infinity, equipped with a degasser G4225A, a binary pump G1312B, an autosampler G1329B, a thermostatic column compartment G1316A, and coupled with three detectors: a multi-channel UV–Vis spectrophotometric detector (DAD) G4212A, a spectrofluorimetric detector (FLD) G1321B, and a spectrometer 6420 Triple Quad LC/MS. The column used was a reversed phase C18, Zorbax SB-C18 (dimensions 2.1 × 50 mm, particle size 1.8 μm).

Briefly, the method involves (i) direct injection of a small volume of emulsion without preliminary treatments; (ii) chromatographic separation of the hydrocarbons from the surfactant fraction; (iii) integration of the chromatographic peaks obtained with the fluorescence detector. Several DMA/surfactant dispersions containing the same amount of surfactant and different DMA concentrations that covered the concentration range of adsorption experiments were used for instrumental calibration.

The method settings were as follows: sample injection volume 2 μL; eluent flow rate 0.6 mL min^−1^; column temperature 30 °C; eluent composition: water with 0.1% formic acid/acetonitrile (35%:65%); excitation wavelength 240 nm; emission wavelength 350 nm; signal integration: 1.1–7 min and 0.8–8 min for DMA/SDBS and DMA/SLS dispersions, respectively.

FLD is the same detector used in many OWS loaded on ships and the results reliability was based on the assumption that the ratio between fluorescent and non-fluorescent hydrocarbons fractions remains constant during the purification (adsorption in our case) treatments.

To verify this assumption, the equilibrium hydrocarbons concentration in a series of dispersions after adsorption onto one of the adsorbent materials investigated was also measured. For this purpose, we performed a solvent extraction of the hydrocarbon fraction followed by the analysis of the extract as reported by other traditional methods commonly adopted for aqueous dispersions analysis (UNI EN ISO 9377–2 2002; APAT / IRSA-CNR 2003; US EPA 2003a, b, c, 2014).

The TPH determinations were also performed by extracting hydrocarbons with hexane, accordingly to the EPA method 1664a (US EPA 1999). To reduce surfactant interference during the liquid–liquid extraction, the procedure has been modified as follows: about 5.00 g of dispersion (weighed on a technical balance Sartorius TE412) were posed into a threaded 12 mL test tube. Then, 6 µL of a solution of perdeuterated eicosane at the concentration of 100 mg L^−1^ were added as an internal standard (IS) to increase accuracy and measure the method recovery. Finally, were rapidly added 0.7 g of KCl, 0.5 g of CaCl_2_ anhydrous, about 20 mg (a single pearl) of reagent grade KOH and 3 mL of hexane. The test tube was then closed with a PTFE sealing cap to avoid any volatile compound loss. The test tube was gently shaken with hands to allow the dissolution of the KOH pearl, and vigorously shaken in a vortex mixer for 60 s to allow the complete and intimate mixing of the aqueous and organic layers. The dispersion obtained was centrifuged for 5 min at 3500 rpm on a Thermo Fisher SL16 centrifuge to separate the phases. After the centrifugation step about 2.5 mL of the superior organic layer have been carefully taken (by a glass Pasteur pipet) from the test tube and treated with 400 mg of anhydrous granular sodium sulfate to remove the residual water. About 1.0 mL of the resulting anhydrous solutions have been deposed in vials and analyzed on a Thermo Fisher Trace GC 1300, equipped with a programmable temperature vaporizer (PTV), and coupled with a Thermo Fisher TSQ8000 triple quadrupole mass spectrometer.

The instrumental parameters used were the following: GC column: poly(dimethyl siloxane), Supelco SPB-1, 30 m length, 0.25 mm I.D.; df 0.25 μm. PTV injector parameters: PTV mode = splitless; splitless time 2 min; split flow 12 mL min^−1^; septum purge flow 5 mL min^−1^; injection temperature = 60 °C, injection time = 0.05 min, evaporation temperature = 60 °C, evaporation time = 0.5 min; transfer temperature = 320 °C; ramp rate = 14.5 °C s^−^ ^1^; transfer time = 2 min; cleaning temperature = 320 °C; time 10 min at 50 mL min^−1^ flow rate, carrier gas helium at 99,9995% purity; gas flow program 1 mL min^−1^ held for 14.5 min switch to 1.5 mL min^−1^ held for 5 min.

GC oven program parameters were the following: initial temperature 80 °C, held for 2 min, thermal gradient at 20 °C min^−1^ up to 310 °C, held for 5 min.

The mass spectrometer, equipped with an EI source that was set with an ionization potential of 70 eV, operated in SIM mode monitoring the ions with the following m/z: 55, 57, 66, 91; additionally, the MS/MS transitions with m/z 85 → 43 (with a collision energy of 10 eV) and m/z 98 → 50 (with a collision energy of 12 eV) were followed, to reduce matrix interferences and increase sensitivity for both TPH and IS detection.

Collision energies and MS/MS transitions were chosen and optimized adopting the automated SRM software available on the spectrometer.

Sample quantitation was performed using a five-point calibration curve in the range of 10–200 mg L^−1^. Standard solutions were prepared in hexane, and each solution contained the IS (perdeuterated eicosane) at 200 μg L^−1^.

Sample recovery was performed on the deuterated internal standard fortifying the matrix (tap water added with SDBS 0.25%) with various levels of hydrocarbons and a fixed level (200 μg L^−1^) of internal standard. An example of the obtained chromatograms is shown in the “Supplementary Materials” (Fig. SM2).

The average recovery on spiked matrix samples, calculated according to the EPA 1664a method (US EPA 1999) was 94% (74–114%) and fell within the suggested acceptance criteria therein indicated.

### Batch adsorption experiments by TOC measurements

Preliminary batch isotherm adsorption experiments were carried out with dispersions containing DMA and surfactant and choosing one of the adsorbent materials investigated, by using the TOC technique. The organic carbon measured in the dispersions is attributable to the sum of carbon coming from both the hydrocarbons and the surfactant. For this reason, two isotherm experiments were carried out as follows: different amounts of ABCP (15–300 mg) were placed in Erlenmeyer flasks containing 25 mL of dispersions of (i) DMA and SLS (*c*_*DMA*_ = 200 mg L^−1^, *c*_*SLS*_ = 500 mg L^−1^) and (ii) only SLS (*c*_SLS_ = 500 mg L^−1^), at pH = 7.0 and at *T* = 25 °C. The samples were shaken for 24 h (the reaching of adsorption equilibrium was previously verified) using an orbital shaker, filtered, and then analyzed employing a TOC apparatus (Shimadzu TOC-5050). TOC has been previously calibrated by using two sets of standard solutions/dispersions containing (i) different SLS concentrations and (ii) 500 mg L^−1^ of SLS and different DMA concentrations (10–100 mg L^−1^).

### Batch and column adsorption experiments by HPLC-FLD measurements

Isotherm experiments of hydrocarbons adsorption onto the four adsorbent materials investigated (FS400, BCP, ABCP, and BBCP) were carried out in batches, in aqueous dispersions containing DMA (*c*_DMA_ ≈ 200 mg L^−1^), SLS (*c*_SLS_ = 6 g L^−1^) or SDBS (*c*_SDBS_ = 2.5 g L^−1^) (surfactants concentrations were always higher than their CMC), without or with the addition of NaCl (0.1 ≤ *c*_NaCl_ (mol L^−1^) ≤ 0.5), at pH = 7 and 7.5 in SLS and SDBS, respectively and at *T* = 25 °C. For each isotherm experiment, different amounts of adsorbent (3–500 mg), were placed in Erlenmeyer flasks containing 20 mL of dispersion at fixed experimental conditions and shaken for 24 h (the reaching of adsorption equilibrium was previously verified) using an orbital shaker (Bernareggio Instruments s.r.l.).

Additionally, to exclude a possible effect of pH on the adsorption abilities of the adsorbents towards DMA, the dependence of the adsorption ability of BBCP, the best adsorbent of DMA, on the pH was checked in single batches. More in details, 4.4 mg of BBCP were placed in four Erlenmeyer flasks together with 20 mL of aqueous dispersions containing DMA (*c*_DMA_ ≈ 200 mg L^−1^), SDBS (*c*_SDBS_ = 2.5 g L^−1^), at different pH values (4, 6, 7.5, and 9) and at *T* = 25 °C. The suspensions were shaken for 24 h.

The hydrocarbons concentrations in the aqueous dispersions, before and after adsorption, were measured after filtration. Measurements were done employing the HPLC-FLD technique (see “Hydrocarbons and hydrocarbons-surfactant quantification methods” for method details). The reliability of the results obtained with the HPLC-FLD technique was previously verified by analyzing the TPH of some post-adsorption dispersions on FS400 also with the GC–MS-MS technique.

In more detail, three different amounts of FS400 were placed in Erlenmeyer flasks with 30 mL of a water dispersion containing DMA 160 mg L^−1^ and SDBS 2.5 g L^−1^ at pH = 7.5 and at *T* = 25 °C. The collected samples, in duplicate, were shaken for 24 h, filtered and then were analyzed both with the HPLC-FLD and GC–MS-MS techniques.

**Fig.1:**
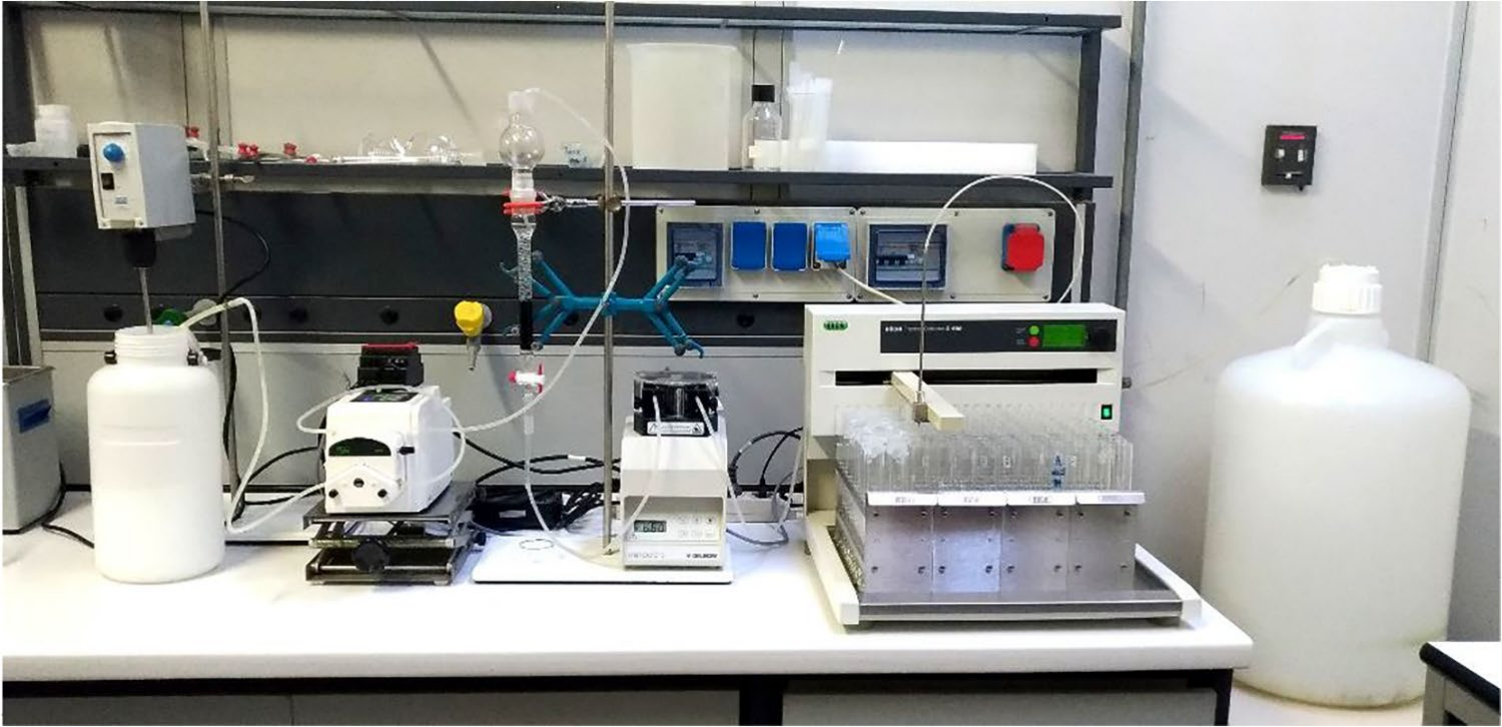
Benchtop pilot system for the removal of hydrocarbons from synthetic bilge waters

Fixed-bed column experiments were also conducted in a laboratory‐scale bench pilot system (Fig. 1).

The system consists of (i) a tank for synthetic bilge water kept in continuous agitation; (ii) a peristaltic pump (Dulabo Laborgerate, mod. PLP 380) to flow the dispersion to the column; (iii) a glass column with an inner diameter of 1.5 cm and length 18 cm filled with the adsorbent material; (iv) a peristaltic pump (Gilson, mod. Minipuls 3) to flow the spilled dispersion and maintain a constant flow rate; (v) a 120-position fraction collector (BUCHI, mod. C-660), and (vi) a discharge tank. The adsorbent bed was packed in between a glass membrane and glass beads. Glass membrane was used as a support to the adsorbent bed and glass beads to avoid the dispersion and the rising of the adsorbent material in the dispersion. Distilled water was passed through the column to withdraw the air trapped between the particles. The dispersion was pumped through the adsorbent bed in down‐flow mode using two peristaltic pumps: the first pump to make the dispersion flow from the container to the head of the column, the second downstream of the column. Two column experiments have been carried out with ABCP (1 g) and BBCP (0.3 g) adsorbents and with dispersions containing DMA (300 mg L^−1^) and SLS (6 g L^−1^) or SBDS (2.5 g L^−1^). The dispersion spilled from the column was collected at specific time intervals in glass tubes by using the fraction collector. Then, hydrocarbons concentration in collected samples was measured by the HPLC-FLD technique.

### Models for equilibrium adsorption studies

The well-known isotherm models of Freundlich (Eq. 1), Langmuir (Eq. 2), and Sips (Eq. 3) (Freundlich 1906; Langmuir 1918; Sips 1948) were used to fit the adsorption equilibrium data:

1 $${q}_{e}={K}_{F}{c}_{e}^{1/n}$$

2 $${q}_{e}=\frac{{q}_{m}{K}_{L}{c}_{e}}{1+{K}_{L}{c}_{e}}$$

3 $${q}_{e}=\frac{{q}_{m}{K}_{S}{c}_{e}^{1/s}}{1+{K}_{S}{c}_{e}^{1/s}}$$

where *q*_*m*_ is the maximum adsorption capacity of the adsorbent material in mg g^−1^; *n* and *s* are empirical parameters related to the strength of adsorption; *K*_*F*_, *K*_*L*_, and *K*_*S*_ are the Freundlich (L^1/*n*^ g^−1^ mg^1−1/*n*^), Langmuir (L mg^−1^), and Sips (L^1/s^ mg^−1/s^) constants and their values give information on the affinity of the adsorbent towards the hydrocarbons. *c*_e_ (mg L^−1^) is the hydrocarbons concentration in the dispersion at the adsorption equilibrium. According to the Freundlich model, the adsorption involves heterogeneous sites of the adsorbent, while the Langmuir model describes the adsorption as a process in which equivalent sites are involved and the adsorbent can be saturated obtaining a monolayer. Both adsorption processes are contemplated in the Sips model.

The equilibrium adsorption capacity at different hydrocarbons/adsorbent ratios (*q*_*e*_, mg g^−1^) has been calculated by the Eq. 4:

4 $${q}_{e}= \frac{V({c}_{0}-{c}_{e})}{m}$$

where *V*(L) is the volume of the dispersion, *m* is the mass of adsorbent material (g), *c*_*0*_ and *c*_*e*_ are the initial and the equilibrium hydrocarbons concentrations in the dispersions (mg L^−1^).

## Results and discussion

### Biochars characterization

The BCP was produced for pyrolysis of dead *Posidonia oceanica* at 400 °C. The BCP yield was 33.2 wt % (see ref. (Cataldo et al. 2018) for more details).

The elemental analysis of the adsorbent materials is reported in Table 1 together with the elemental analysis of the dead *Posidonia oceanica*. Both pyrolysis and chemical activations processes cause an increase in the carbon percentage which nearly doubles in the case of BBCP (from 46.14 to 85.57 wt %) and a decrease in O/C ratio (0.64 and 0.09 for BCP and BBCP, respectively) used to monitor the carbonization process. These trends can be attributable to the volatilization of oxygenated compounds (Grierson et al. 2009; Cataldo et al. 2018). Taking into consideration the literature values of C and O/C wt % reported for FS400, this commercial activated carbon looks similar to the BBCP.

**Table 1:** Results of elemental analysis

Adsorbent	C	H	N	S	O	O/C	Reference
*Posidonia oceanica*	46.14	6.82	1.28	0.33	29.73	0.64	(Cataldo et al. 2018)
BCP	49.54	2.41	1.52	0.08	28.00	0.56	(Cataldo et al. 2018)
ABCP	63.66	3.23	2.03	0.21	12.43	0.19	(Cataldo et al. 2018)
BBCP	85.57	2.31	1.62	-	7.92	0.09	This work
FS400	87.50	0.18	0.80	0.65	10.87	0.12	(Rivera-Utrilla and Sánchez-Polo 2002)

Dry basis ± 0.02

Data of N_2_ adsorption–desorption measurements including surface area, micropore surface, pore volume and pore width are reported in Table 2. Adsorption isotherm curves at − 196 °C and pore size distribution for both BCP and BBCP are reported in Figs. SM3 and SM4 of the Supplementary Materials, respectively.

**Table 2:** Nitrogen adsorption–desorption measurements

N_2_ adsorption–desorption measurements				
	**BCP**^**a)**^	**ABCP**^**a)**^	**BBCP**	**FS400**^**b)**^
BET m^2^ g^−1^	4.664	20.936	650.8^c)^	800–1200
T-plot micropore area m^2^ g^−1^	2.378	5.726	484.85^c)^	636–1045
Desorption average pore width (4 V/A) nm	12.905	3.324	2.791	-
Pore Volume cm^3^ g^−1^	0.0150	0.0174	0.454	0.49–0.70

^a)^Ref. (Cataldo et al. 2018); ^b)^Ref. (Morlay and Joly 2010); ^c)^ ± 3.4%

The chemical activation of BCP led to a change in surface area, pore volume, and average pore width. In particular, for the basic activation the surface area and pore volume increase from 4.664 to 650.8 m^2^/g and from 0.0150 to 0.454 cm^3^ g^−1^, respectively. On the other side, the average pore width decreases from 12.905 to 2.791 nm.

The high surface area value (650.8 m^2^ g^−1^) recorded for BBCP sample (see Fig. SM3), is mainly derived from micropores presence as revealed by the high value of micropore area (484.85 m^2^ g^−1^); this is evident from the form of the isotherm that is of type I, typically attributed according to the IUPAC classification at the microporous solids (Rouquerol et al. 1994).

Feature of this type of isotherms, as shown in Fig. SM3 b, is a rapid increase in the adsorption due to micropores filling at very low relative pressures starting at P/ P_0_ below10^−5^ and upon reaching a plateau at relative pressure values higher than *P*/*P*_0_ = 0.3, corresponding to monolayer accommodation and saturation with adsorbed N_2_ on the external surface (Rouquerol et al. 1994).

In the current case, the micropores surface area represents about 74% of the BET surface area (Table 2), indicating that the activation method was able to induce the creation of a microporous material as also confirmed by results reported in Fig. SM4. In particular, as depicted in Fig. SM4, the activation treatment deeply influenced the pore size distribution of BBCP. An evident shift of pore distribution from meso/macro structures to microporous structures for the activated biochar was observed. This higher amount of micropores and then the development of porosity obtained from activation with KOH can be attributed to the enlargement of the graphitic planes interlayer space due to potassium intercalation and deintercalation (Wang and Kaskel 2012). The FS400 data highlight the high surface area of this commercial activated carbon which is the material with the highest surface area among those here considered.

**Fig. 2:**
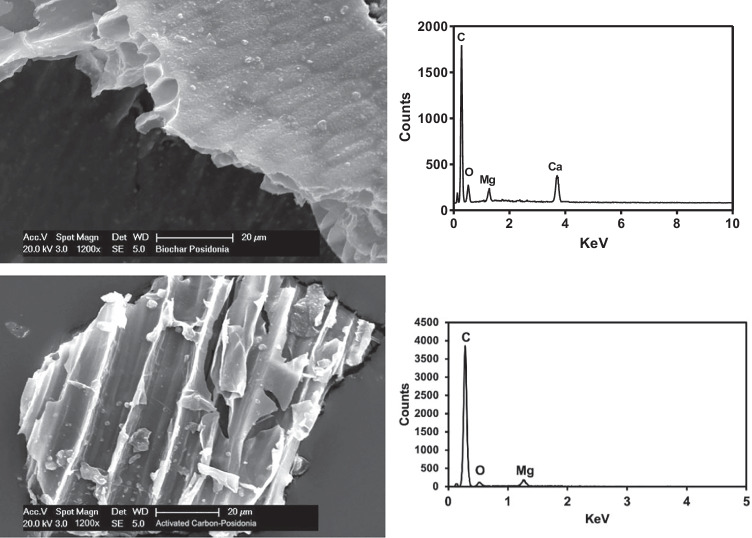
SEM micrographs and EDX spectra of BCP (top), BBCP (bottom) at 1200 × magnification

To better investigate the morphology, elemental composition and texture of the biochars, SEM analysis coupled with EDX characterization was performed. Since the SEM–EDX results of BCP and ABCP were discussed elsewhere (Cataldo et al. 2018), we focus here on the comparison between BCP and BBCP. Figure 2 shows the surface of BCP and BBCP samples at the same magnifications (1200 ×).

The surface of BCP appears homogeneous and without holes while it changes after the chemical activation with modification of the porous structure. It is clear that the activation methodology enhanced the development of the porosity at different levels and in particular, it has been observed that the striped texture present in the inner walls of the BBCP enhances the overall surface area and therefore the adsorption properties (Ncibi et al. 2009).

EDX spectra of BCP and BBCP samples evidenced an increase in carbon and a decrease in oxygen (see Table 3), in agreement with the results from the elemental composition reported in Table 1, and confirmed the carbonization process promoted by the activation treatment. Furthermore, due to the cleaning effect of potassium hydroxide treatment, the chemical activation also lowers the Ca and Mg concentrations in BBCP (due to calcium carbonate deposits, see Fig. SM5). Literature EDX microanalysis for FS400 shows a carbon/ oxygen content similar to BCP (or ABCP), despite the high difference in the surface area of these materials.

**Table 3:** EDX microanalysis

Elements	BCP at%^a)^	ABCP at%^a)^	BBCP at%	FS400 at%^c)^
C	83.43	85.52	95.76^b)^	85
O	12.76	9.94	3.37^b)^	11.5
Mg	1.30	0.81	0.78^b)^	
Ca	3.21	2.51	-	
Al	-	-	-	1.5
Si	-	-	-	2

^a)^Ref. (Cataldo et al. 2018); ^b)^ ± 5%; ^c)^Ref. (Gensterblum et al. 2009)

**Fig. 3:**
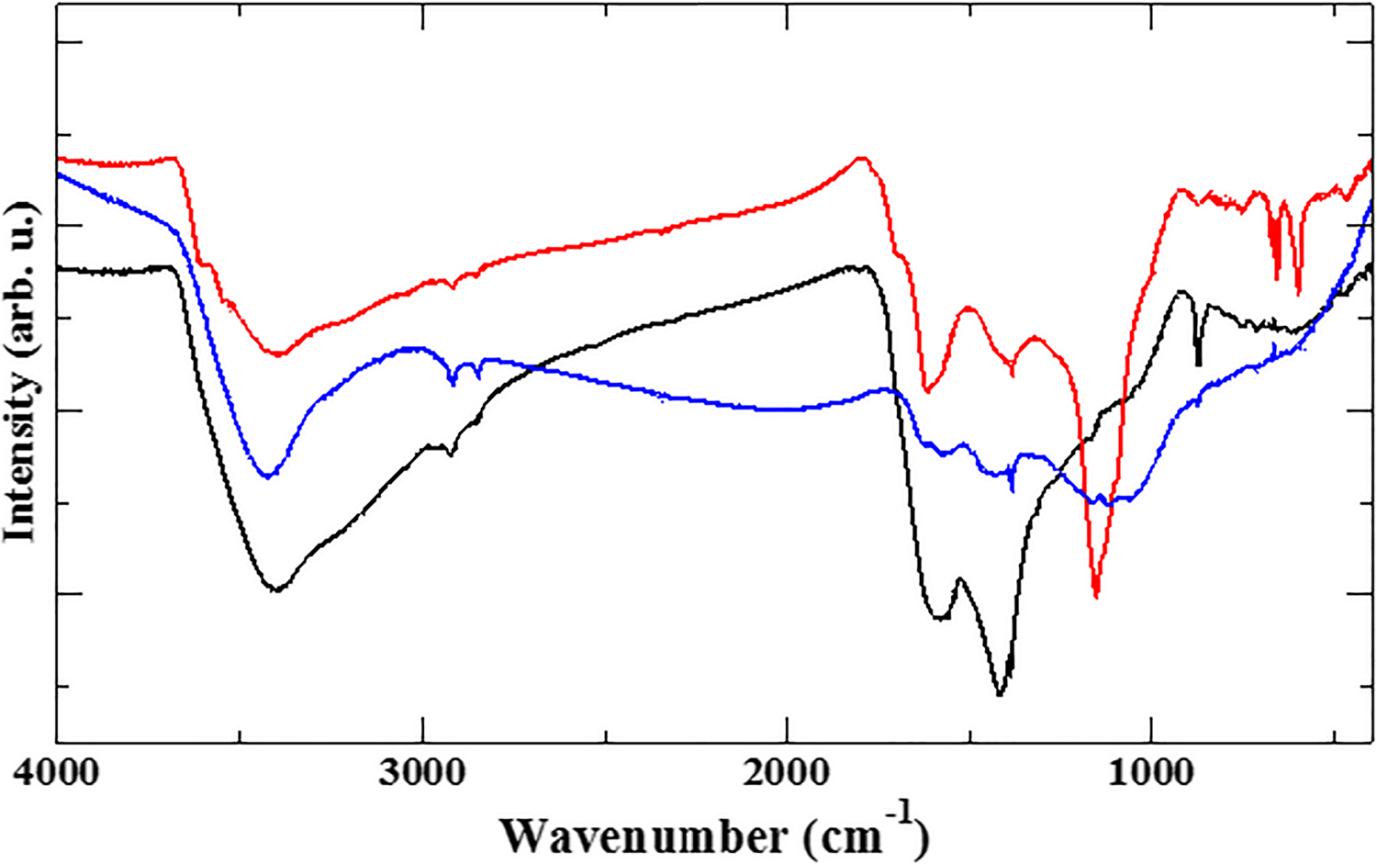
Infrared absorption spectra (shifted vertically for clarity) of BCP (black), ABCP (red), and BBCP (blue)

The activation treatments also induce deep chemical modifications on the surface of the carbonaceous particles, as is evident from both infrared spectra and thermogravimetry. Differential thermogravimetric curves (see Fig. SM6) show that BCP thermally degrades with the main peak at 500 °C, and a further minor mass loss at 620 °C. On the contrary, both ABCP and BBCP show one single mass loss, and significantly higher thermal stability compared to BCP. In particular, the BBCP degradation peaks at about 550 °C, and ABCP shows a broader peak at 550–600 °C. The infrared spectra are shown in Fig. 3. Some of the main features of the spectra are maintained after the treatments: the broad O–H stretching around 3400 cm^−1^, two sharp aliphatic C-H stretching around 3000 cm^−1^, C = C stretching around 1600 cm^−1^, and the band around 1400 cm^−1^ which could be due to either = CH_2_ or O–H bending. The ABCP sample shows the most striking changes in the spectrum, starting with the sharp peak around 3600 cm^−1^, which is indicative of free O–H not hydrogen-bonded to other oxygen atoms. A very intense band at 1155 cm^−1^ is due to C-O stretching: both these bands hint at the formation of C-O–H surface species. This latter band, but less definite and less intense, is also present in the spectrum of BBCP.

Water contact angle measurements carried out onto dried pellets of BBCP as it was and after contact with SLS and SDBS solutions (see “Preparation and characterization of biochars” for experimental details) showed that, after loading with surfactant, the contact angle *θ*_*i*_ decreases from 34.4° to 22.5° and 20.6° for SLS and SDBS loading, respectively. The increased hydrophilicity of the adsorbent surface caused by surfactants adsorption suggests that it occurs preferentially through the hydrocarbon tails with the polar heads of surfactants facing the aqueous phase. This agrees with the increase in wettability of carbonaceous materials promoted by the adsorption of surfactants highlighted by molecular dynamics studies (Liu et al. 2020; Jin et al. 2022).

### Preliminary batch adsorption experiments carried out through TOC measurements and reliability of HPLC-FLD measurements

One of the objectives of this work is to find a suitable instrumental technique to rapidly quantify the hydrocarbons present in an aqueous dispersion comparable to bilge waters. To this end, two sets of standard aqueous solutions/dispersions containing only SLS and SLS and DMA have been prepared (see “Batch adsorption experiments by TOC measurements” for details) and used to calibrate the TOC instrument. Good linearity and a suitable sensitivity have been obtained in the concentration range investigated and TOC technique has been used to carry out the batch adsorption experiments of DMA–SLS dispersions onto ABCP material.

Considering that TOC represents the sum of the organic carbon derived from both surfactant and hydrocarbons in the dispersions, two adsorption isotherm experiments with the ABCP have been done with dispersions containing only SLS and both DMA and SLS (see “Batch adsorption experiments by TOC measurements” for experimental details) to be able to discriminate the adsorption of the surfactant from that of hydrocarbons.

Experimental data have been tentatively fitted with the three isotherm models and the best model in terms of experimental data fit was the Sips equation (highest *R*^2^ and lowest std. dev. of the fit).

**Fig. 4:**
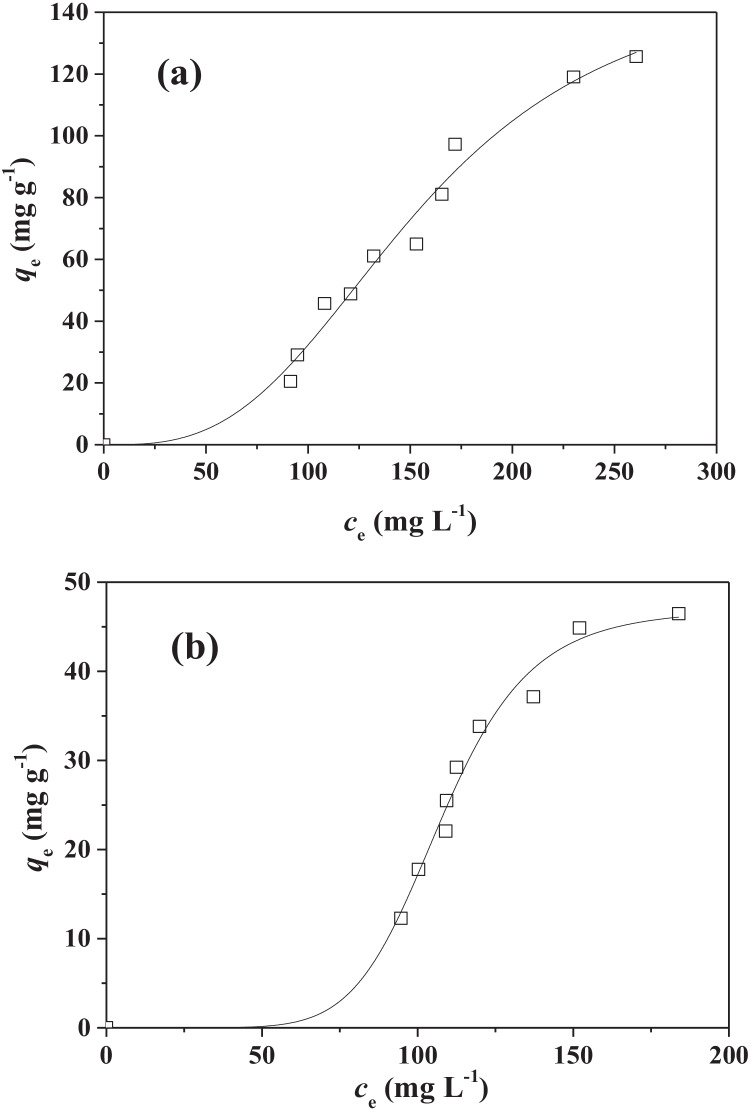
Isotherms of DMA and SLS (**a**) and of SLS (**b**) adsorption onto ABCP material obtained by TOC measurements. Experimental data fitted with Sips isotherm equation

Thus, a cooperative mechanism for the adsorption of SLS (see S-shaped curve of the isotherm in Fig. 4b) compatible with the formation of hemi-micelles, as suggested by the results of the contact angle measurements, can take place.

Note that in isotherm experiments carried out by measuring TOC, *c*_*e*_ represents the sum of DMA and surfactant concentrations at adsorption equilibrium expressed in mg of organic carbon per liter of dispersion. Consequently, *q*_*e*_ represents the equilibrium “TOC” adsorption capacity of the material.

The values for the parameters of the Sips equation, the only isotherm model able to fit the experimental data, are reported in Table 4 together with errors and statistical parameters of fits. The experimental data together with the fit curves of the Sips equation are reported in Fig. 4.

**Table 4:** Sips isotherm parameters for the hydrocarbons and SLS adsorption onto ABCP from dispersions containing DMA (*c*_DMA_ = 200 mg L^−1^) and SLS (*c*_SLS_ = 500 mg L^−1^) or only SLS (*c*_SLS_ = 500 mg L^−1^) at pH = 7.0 and at *T* = 25 °C

Adsorbate	*q*_*m*_ (mg g^−1^)	*K*_*S*_ (L^1/s^ mg^−1/s^)	*s*	*R*^2^
DMA-SLS	153 ± 19	2.6 10^–7^ ± 4.6 10^–7^	0.33 ± 0.04	0.9781
SLS	47 ± 2	7 10^–16^ ± 1.4 10^–15^	0.134 ± 0.008	0.9832

Assuming that the contributions of DMA and SLS are additive for the determination of *q*_*m*_ values, a value of 106 ± 21 mg of TOC per g of ABCP can be estimated for only DMA adsorption.

Although preliminary TOC measurements suggest that DMA is significantly adsorbed by ABCP, it must be underlined that it is only a rough estimate because it is not possible to quantitatively distinguish the part attributable to DMA without assumptions. Therefore, an alternative equally fast method able to selectively quantify the hydrocarbons in the aqueous dispersions has been tested by measuring the hydrocarbons concentration directly in the dispersions through the HPLC-FLD technique (see “Hydrocarbons and hydrocarbons-surfactant quantification methods”). This method does not provide for the preliminary solvent extraction steps of traditional methods and allows the treatment of a large number of samples in a reasonably short time. Before carrying out the systematic study of the adsorption ability of the four adsorbent materials investigated, the reliability of the results obtained with this instrumental technique was verified by analyzing the TPH of a series of post-adsorption dispersions on the commercial activated carbon FS400 also with the GC–MS-MS technique (see “Hydrocarbons and hydrocarbons-surfactant quantification methods” and “Batch and column adsorption experiments by HPLC-FLD measurements” for method and experimental details, respectively).

A comparison of the equilibrium hydrocarbons concentrations (*c*_*e*_) measured by HPLC-FLD and GC–MS-MS in aqueous dispersion (DMA 170 mg L^−1^ and SDBS 2.5 g L^−1^) after adsorption onto three different amounts of FS400 is reported in Table 5. Considering the experimental errors in the concentration values, the results obtained with the two instrumental techniques are comparable and confirm the reliability of HPLC-FLD measurements.

**Table 5:** Equilibrium hydrocarbons concentrations (*c*_*e*_) measured in a dispersion (*c*_*DMA*_ = 170 mg L^−1^, *c*_*SDBS*_ = 2.5 g L^−1^, at pH = 7.5 and at *T* = 25 °C) after contact with different amounts of FS400 by HPLC-FLD and GC–MS-MS techniques

FS400 (mg)	*c*_*e*_ (mg L^−1^)	
	HPLC-FLD	GC–MS-MS
10.7	112 ± 2	114 ± 8
13.4	79 ± 2	72 ± 9
16.1	62 ± 2	49 ± 6

### Modeling of equilibria of hydrocarbons adsorption onto BCP, ABCP, BBCP, and FS400 by HPLC-FLD technique

Adsorption equilibria of hydrocarbons onto BCP, its activated forms ABCP and BBCP and the commercial activated carbon FS400 have been studied by using aqueous dispersions that simulated bilge waters, containing the DMA, a surfactant at a concentration higher than its CMC (SLS or SDBS), and NaCl at different concentrations (see “Batch and column adsorption experiments by HPLC-FLD measurements” for experimental details).

The experimental data *q*_*e*_ vs. *c*_*e*_ were fitted with Langmuir, Freundlich, and Sips equations. Independently of the experimental conditions and of the adsorbent material used in the experiments, among the tested isotherm models, the Langmuir equation was the best or it was equivalent to the Freundlich equation in terms of quality of fits. The data fits with the Sips model were the worst, did not converge, or, at best, gave results equivalent to Langmuir’s model (*s* ≈ 1). For this reason, only values for Langmuir parameters are reported in the manuscript. The parameters values of all the other tested isotherm equations are reported in Tables SM1 – SM3 of Supplementary Material.

**Fig. 5:**
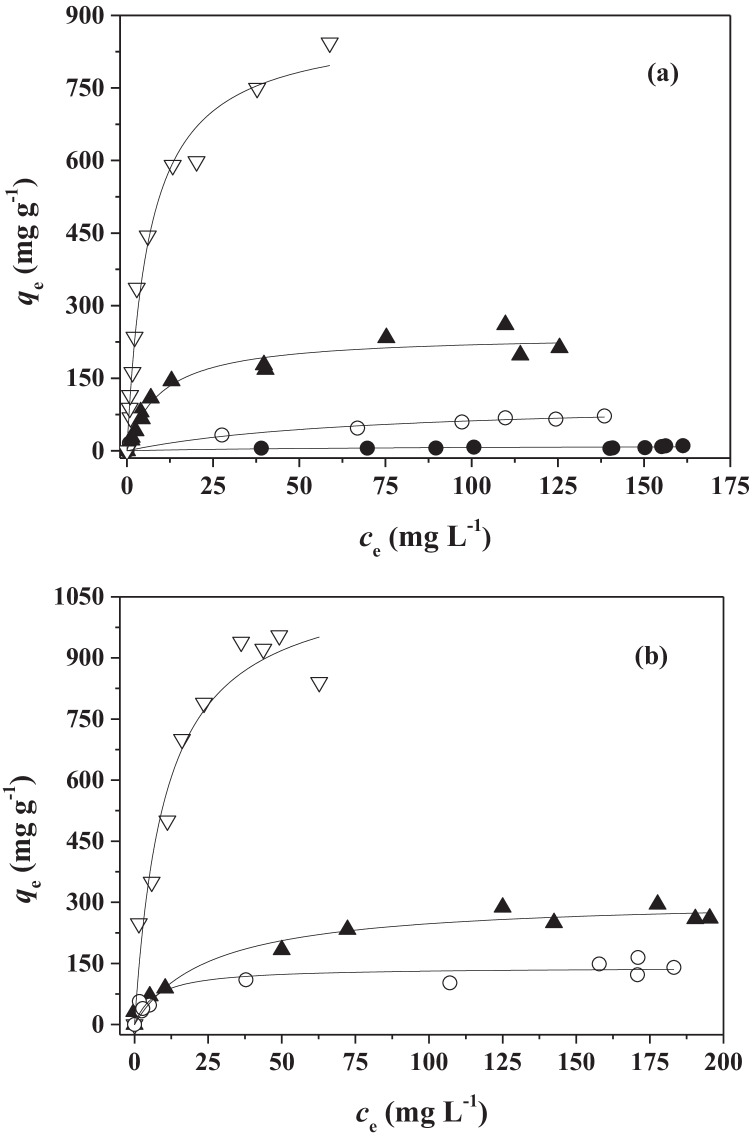
Adsorption isotherms of hydrocarbons onto BCP (●), ABCP (○), BBCP (∇), and FS400 (▲) from aqueous dispersions of DMA (*c*_DMA_ = 200 mg L^−1^) and SLS (*c*_SLS_ = 6 g L^−1^) (**a**) or SDBS (*c*_SDBS_ = 2.5 g L^−1^) (**b**) pH = 7 and 7.5 in SLS and SDBS, respectively and at *T* = 25 °C. Experimental data fitted with Langmuir equation

At first, the hydrocarbons adsorption abilities of all adsorbents were studied towards dispersions containing DMA (*c*_DMA_ = 200 mg L^−1^) and SLS (*c*_SLS_ = 6 g L^−1^). The values for Langmuir equation parameters are reported in Table 6 and the experimental data together with the curve fit of the isotherm model are depicted in Fig. 5a.

**Table 6:** Langmuir isotherm parameters for the hydrocarbons adsorption onto BCP, ABCP, BBCP, and FS400 from dispersions containing DMA (*c*_DMA_ 200 mg L^−1^), SLS (*c*_SLS_ 6 g L^−1^), or SDBS (*c*_SDBS_ 2.5 g L^−1^), NaCl (0 ≤ *c*_NaCl_/mol L^−1^ ≤ 0.50) at pH = 7 and 7.5 in SLS and SDBS, respectively and at *T* = 25 °C

Adsorbent	Surfactant	NaCl	*q*_*m exp*_ (mg g^−1^)^b^	*q*_*m*_ (mg g^−1^)	*K*_*L*_ (L mg^−1^)	*R*^2^
BCP	SLS	0 ^a^	11	11 ± 4	0.014 ± 0.014	0.7518
ABCP	SLS	0	72	104 ± 23	0.014 ± 0.008	0.9364
FS400	SLS	0	213	240 ± 11	0.10 ± 0.02	0.9553
BBCP	SLS	0	843	883 ± 37	0.15 ± 0.02	0.9814
ABCP	SDBS	0	140	140 ± 9	0.14 ± 0.05	0.8789
FS400	SDBS	0	261	309 ± 18	0.04 ± 0.01	0.9640
BBCP	SDBS	0	840	1106 ± 80	0.10 ± 0.02	0.9527
BBCP	SLS	0.10	1044	1182 ± 108	0.05 ± 0.01	0.9523
BBCP	SLS	0.25	1044	1489 ± 140	0.028 ± 0.005	0.9788
BBCP	SLS	0.50	1143	1857 ± 279	0.021 ± 0.006	0.9399

^a^mol L^−1^; ^b^the last experimental *q*_*e*_ value on the right side of the isotherm

The results clearly show that both chemical activations improved the adsorption ability of the biochar, though only by a slight amount in the case of acidic activation. Instead, basic activation increases the hydrocarbons uptake of BCP 80-folds.

It is noteworthy that, although the *q*_*m*_ value obtained for ABCP (*q*_*m*_ = 106 mg g^−1^) from the adsorption isotherms made by the TOC technique was only an estimate, it is comparable with the *q*_*m*_ value of the reliable HPLC-FLD measurements (*q*_*m*_ = 104 mg g^−1^).

The adsorption capacity trend (BBCP >  > FS400 > ABCP > BCP) may seem, in some ways, unexpected considering that the material with the highest surface area, i.e., FS400, (see “Biochars characterization”) does not match the highest *q*_*m*_ value. Apart from some difference in chemical composition between BBCP and FS400 (see Table 1), it can be assumed that the highest adsorption ability of BBCP can be attributable to a different distribution of pores with respect to FS400 as confirmed by the nitrogen adsorption–desorption data reported in Table 2 from which a more open structure with larger pore size is expected for BBCP.

Moreover, FS400 and BBCP have the highest affinity towards hydrocarbons (*K*_*L*_ = 0.10 and 0.15 L mg^−1^, respectively), and this is probably attributable to the more carbonaceous nature of these materials (see Table 1).

The adsorption ability and affinity towards hydrocarbons of the best three adsorbent materials (ABCP, BBCP, and FS400) have been studied also in dispersions containing DMA (*c*_DMA_ = 200 mg L^−1^) and SDBS at a concentration higher than its CMC (*c*_SDBS_ = 2.5 g L^−1^) (see “Batch and column adsorption experiments by HPLC-FLD measurements” for experimental details). The values of Langmuir equation parameters are reported in Table 6 and the experimental data together with the curve fit of the isotherm model are depicted in Fig. 5b.

The adsorption capacities of the adsorbents, in terms of *q*_*m*_ values, are on average higher in SDBS than in SLS, while, the affinity (*K*_*L*_) is similar. This can be justified considering the different structures of the two surfactants and the higher CMC of SLS (2.34 g L^−1^ or, in molar scale, 0.00811 mol L^−1^) with respect to that of SDBS (0.9632 g L^−1^ or, in molar scale, 0.002764 mol L^−1^) (Williams et al. 1955; Yang et al. 2006). Due to the different CMC, the SLS concentration (0.0200 mol L^−1^) used in the adsorption experiments is considerably higher than that of SDBS (0.0072 mol L^−1^). Therefore, considering that all the adsorbent materials can adsorb even the surfactant alone (see results of TOC and of contact angle measurements), the higher SLS concentration probably hinders the adsorption of hydrocarbons.

The experimental data of batch adsorption experiments have been also used to investigate the effect of the adsorbent dosage on their adsorption capacity at equilibrium (*q*_*e*_) and removal efficiency.

The *q*_*e*_ and the removal efficiency values of the four carbonaceous adsorbents vary in the classic way with respect to the adsorbent dosage, i.e., the *q*_*e*_ decreases with the increasing of the adsorbent dosage, while, an opposite trend was found for the removal efficiency. As an example, the effect of adsorbent dosage of BBCP, the best adsorbent among those investigated, on the *q*_*e*_ and on the removal efficiency is reported in Fig. SM7 in DMA–SLS and DMA–SDBS dispersions. In agreement with the data derived from adsorption isotherm, the BBCP dosage required to remove the 100% of hydrocarbons in SDBS is ~ 10 times less than in SLS.

Bilge waters are generally defined as aqueous dispersions of hydrocarbons containing surfactants with an extremely variable percentage of seawater (Peng et al. 2005). The high salinity of seawater could, in some way, positively or negatively affect the ability of the adsorbent material to remove hydrocarbons. For this reason, the effect of salinity on the hydrocarbons uptake of the adsorbent with the highest *q*_*m*_ values (BBCP) was investigated by adding different amounts of NaCl to the DMA/SLS dispersions (see “Batch and column adsorption experiments by HPLC-FLD measurements” for experimental details).

**Fig. 6:**
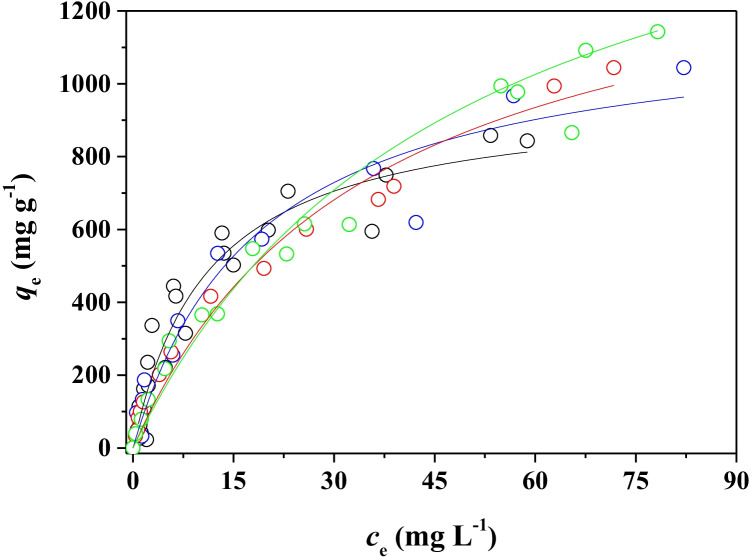
Adsorption isotherms of hydrocarbons onto BBCP from aqueous dispersions of DMA (*c*_DMA_ = 200 mg L^−1^), SLS (*c*_SLS_ = 6.0 g L^−1^), and NaCl at *I* = 0 (black), 0.10 (blue), 0.25 (red), and 0.50 mol L^−1^ (green) at pH = 7.0 and at *T* = 25 °C. Experimental data fitted with Langmuir equation

The values for Langmuir equation parameters at different NaCl concentrations in the range 0–0.50 mol L^−1^ are reported in Table 6, whereas the experimental data together with the curve fit of the Langmuir isotherm are depicted in Fig. 6.

The experimental evidence on the salt effect is quite interesting. It clearly shows that as the salt concentration increases, *q*_*m*_ increases showing at the *I* = 0.50 mol L^−1^ a value more than two times higher with respect to that determined in the absence of NaCl. A reverse trend was observed for the Langmuir constant values. Somehow, the ions of the NaCl salt interact with the surfactant SLS and/or with the adsorbent surface, determining a greater adsorption capacity of the material and at the same time a lower affinity. A possible explanation could be the reduction of the repulsion forces due to the electric double layer of the surfactant/solution interface of the hydrocarbons solubilizing micelles. This effect is well known and is what causes the destabilization of emulsions due to the addition of electrolytes (Hiemenz and Rajagopalan 2017). The adsorbed DMA is highly likely to be incorporated in a hemi-micelle-like structure that favors the hydrophobic interactions of both the DMA and the surfactant with the carbonaceous material. The salt effect on the reduction of repulsion between colloidal particles is plausible and occurs between the adsorbed hemi-micelles since the chemical-physical principles underlying the phenomenon are the same regardless of the geometry of the colloidal particle. Therefore, in the presence of a high salt concentration, the lower repulsion of the hemi-micelles allows a greater adsorbing capacity. On the other hand, the presence of adsorbed surfactant was highlighted for ABCP and appears all the more reasonable for BBCP due to the better adsorption capacity. The results of contact angle measurements corroborate and confirm this hypothesis. Furthermore, the formation of surfactant aggregates at the water/carbonaceous sorbent surface can promote the solubilization of hydrophobic molecules as highlighted by both theoretical and experimental studies (Yang et al. 2010; Liu et al. 2020).

The observed inverse trend for the Langmuir constant values can be attributed to multiple factors including the charge screening effect, competition in adsorption of the electrolyte ions, and effects of the electrolyte on the point of zero charges of the adsorbent material. The clarification of this aspect would merit further investigations which are outside the scope of this work. However, an unfavorable effect of ionic strength on the affinity of the carbonaceous materials towards other adsorbates has also been found in previous works (Cataldo et al. 2016, 2018).

To our knowledge, the effect of salinity on the hydrocarbons adsorption onto activated carbons and biochars has been scarcely investigated (Ji et al. 2020). However, including in the literature search other carbonaceous or clays adsorbent materials and, among the adsorbates, specific categories, or individual hydrocarbons, it can be noted that mixed results have been obtained. The positive or negative effects of salts addition on the hydrocarbons adsorption are usually attributed to the reduction of hydrocarbons solubilities, the changing of physico-chemical properties of the adsorbent, the shielding of repulsive charges between the adsorbent and the oil, and other phenomena such as electrostatic interactions, ion exchange, water adsorption, and the “salting out” effect (Takeuchi et al. 2015; Lamichhane et al. 2016; Diraki et al. 2018, 2019; Ji et al. 2020).

In Table 6 are also reported the *q*_*m* exp_ values, i.e., the last experimental *q*_*e*_ values on the right side of the adsorption isotherms that correspond to the highest *q*_*e*_ values in the range of adsorbent dosage/*c*_DMA_ ratios investigated during the adsorption isotherm studies. In general, the “*q*_*m* exp_” values are in good agreement with the calculated ones except for those at the highest NaCl concentrations. Indeed, the increase of adsorption abilities of BBCP in NaCl 0.30 and 0.50 mol L^−1^ makes the range of adsorbent dosage/*c*_DMA_ ratios investigated not wide enough to approach the maximum adsorption capacity that, in any case, can be extrapolated with a good approximation.

The pH of bilge waters averages in a narrow range (7–9.5) (Church et al. 2019) in which the pH of emulsions used in adsorption experiments falls. However, the effect of pH on the adsorption ability of BBCP towards DMA was studied through adsorption experiments at a single adsorbent dosage/*c*_DMA_ ratio, at pH 4.0, 6.0, 7.5, and 9.0 (see “Batch adsorption experiments by TOC measurements” for experimental details). The *q*_*e*_ values calculated at the different pH values are shown in the histogram of Fig. SM8 of Supplementary Materials. The small differences in *q*_*e*_ values at the four pH values are within the experimental errors confirming that the adsorption capacity of the BBCP is independent of pH.

The knowledge of the isotherm adsorption parameters helps to determine the theoretical operating conditions of a fluidized bed system to remove hydrocarbons from dispersions under the considered experimental conditions, thus predicting the amount of adsorbent material to be used. For example, the treatment of 2 m^3^ of synthetic bilge water with a hydrocarbons concentration of 80 mg L^−1^ needs about 200 g of BBCP to obtain a target hydrocarbons concentration of 15 mg L^−1^.

### Column adsorption experiments results

To simulate the adsorption step of an on-board OWS, fixed-bed column experiments have been finally carried out by using ABCP and BBCP adsorbents and hydrocarbons dispersions containing SLS or SDBS, with a laboratory‐scale bench pilot system (see “Batch and column adsorption experiments by HPLC-FLD measurements” for experimental details).

**Fig. 7:**
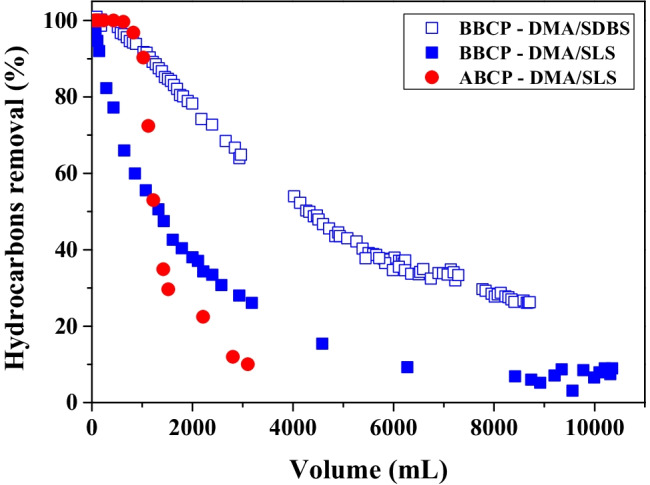
Breakthrough curves of hydrocarbons adsorption onto 1 g of ABCP or 0.3 g of BBCP from aqueous dispersions of DMA (*c*_DMA_ = 300 mg L^−1^) and SLS (*c*_SLS_ = 6.0 g L^−1^) or SDBS (*c*_SDBS_ = 2.5 g L^−1^) at pH = 7 and 7.5 in SLS and SDBS, respectively and at *T* = 25 °C

The results are shown in Fig. 7 through breakthrough curves in which the percent of hydrocarbons removal was calculated as follows:

$$\text{hydrocarbons removal} \, = \text{ } \frac{{\text{c}}_{0}-{\text{c}}_{\text{v}}}{{\text{c}}_{0}}\text{\%}$$

where *c*_*0*_ and *c*_*v*_ represent the initial hydrocarbons concentration and that of the effluent after a given volume of dispersion crossed the column.

The hydrocarbons are initially almost totally adsorbed by both ABCP and BBCP adsorbents. Different breakthrough curve profiles have been found for ABCP and BBCP. The results are in agreement with those of batch experiments, confirming the improved adsorption ability of BBCP. Although the amount of ABCP used in the experiment is more than three times that of BBCP, a 10% hydrocarbons removal was found after only 3000 mL of dispersion. With a dispersion containing the same hydrocarbons and SLS concentrations (*c*_DMA_ = 300 mg L^−1^; *c*_SLS_ = 6.0 g L^−1^), the same removal % was reached after ~ 6000 mL.

Only for BBCP, the same column experiment was done using the surfactant SDBS. A comparison of the breakthrough curves of hydrocarbons adsorption onto BBCP with the dispersions containing SLS and SDBS shows an improvement of adsorption performances of BBCP with the dispersion containing the latter surfactant, with hydrocarbons removal percent higher than 20 also after the spillage of 9000 mL of effluent. Once again, the outcome of column experiment confirms the results obtained through the batch adsorption isotherm.

In general, the adsorbent material was considered exhausted when a removal rate of 20% was reached.

### Literature data comparison

The ability of an adsorbent material to remove hydrocarbons from an aqueous dispersion depends on a large number of variables such as (i) the composition of the hydrocarbons mixture used in the experiments, e.g., the marine gas oil DMA like in our case, other petroleum-based fuels, oils, aromatic hydrocarbons, polycyclic aromatic hydrocarbons (PAHs); (ii) if used, the type and the concentration of surfactant and if it is below or above its CMC; (iii) the salinity of the aqueous dispersion. However, a rough comparison of the adsorption abilities of biochars here investigated with some of the most studied carbonaceous adsorbents reported in the literature can be done (Sueyoshi et al. 2012; Emam 2014; Ji et al. 2020; Yousef et al. 2020).

Sueyoshi et al. (Sueyoshi et al. 2012) reported adsorption capacities of 1330 and 1425 and 730 mg g^−1^ for the hydrocarbons adsorption onto two steam activated carbons of date palm trunk (*Phoenix dactylifera*) and a commercial coconut shell-based activated carbon, from heavy oil emulsions stabilized respectively, with 7–10 mg L^−1^ of SLS. The adsorption data were extrapolated from breakthrough curves and, considering the higher SLS and SDBS concentrations of our experiments (6.0 and 2.5 g L^−1^, respectively) are comparable with those here obtained for BBCP.

A commercial activated carbon has been tested by Emam (Emam 2014), as it was (NRS) and after a chemical activation in HNO_3_ 1 mol L^−1^ (NA-NRS), as adsorbent of hydrocarbons from dispersions containing a diesel oil and a not specified amount of the surfactant Triton X-100. Except for BCP, the adsorption abilities of both NRS and NA-NRS adsorbents (*q*_*m*_ = 31.25 and 65.79, respectively) were much lower than those of the biochars here investigated.

Ji et al. studied the hydrocarbons adsorption onto three commercial activated charcoals, two granular (GAC4X12 and GAC12X20) and one powder (PAC100), from two types of water mixtures containing oil and dispersant (Corexit EC9500A or Corexit EC9527A). The two dispersants were a mix of solvents and surfactants usually used during oil spills. Looking at the constant values of Freundlich isotherm model (*K*_*F*_ = 0.003, 0.467 and 10.898 L^1/*n*^ g^−1^ mg^1–1/*n*^) reported by the authors, the three charcoals have lower adsorption abilities towards hydrocarbons than the ABCP, BBCP, and FS400 here investigated. The authors also studied the effect of surfactant concentration on the hydrocarbons adsorption of charcoals. In particular, below the CMC, they found that the addition of surfactant facilitates adsorption. On the contrary, above the CMC, the hydrocarbons solubilization prevails over the adsolubilization phenomena reducing the adsorption ability of the adsorbent (Ji et al. 2020).

Considering that the adsorption studies done in this work were conducted in presence of SLS or SDBS at concentrations higher than their CMC the materials studied here should likely perform even better at lower surfactant concentrations as hypothesized in previous sections.

## Conclusion

The hydrocarbons removal from synthetic bilge waters (water dispersions containing the marine gas oil DMA, a surfactant and different amounts of NaCl) through the adsorption onto biochars, pristine, or chemically activated, coming from pyrolysis of dead *Posidonia oceanica* have been studied carrying out numerous batch and column adsorption experiments. Several instrumental techniques have been tested to find a quick and accurate way to measure the hydrocarbons concentrations in the collected aqueous dispersions before and after the adsorption onto the adsorbent materials. The pristine and the activated biochars have been characterized through elemental analysis, nitrogen adsorption–desorption analysis, SEM–EDX microanalysis, FT-IR spectroscopy, TGA, and water contact angle measurements. The main results of this study can be summarized as follow:

- The HPLC-FLD technique was suitable for measuring the concentration of hydrocarbons directly in dispersions, avoiding the time-consuming matrix pre-treatment steps typical of common standardized methods.
- The adsorption capacity trend of the adsorbent materials investigated was BBCP >  > FS400 > ABCP > BCP. The highest hydrocarbons uptake of BBCP with respect to the other biochars was ascribed to its more carbonaceous nature and to a more open structure with a larger pore size as evidenced by the nitrogen adsorption–desorption data.
- The surfactants, added to the dispersions, at a concentration higher than their CMC, play an important role in hydrocarbons adsorption. In particular, the adsorption capacities of the adsorbents, in terms of *q*_*m*_ values, were on average higher in SDBS than in SLS and it has been attributed to the higher concentration of SLS than that of SDBS used in the experiments (0.0200 and 0.0072 mol L^−1^, respectively).
- The effect of ionic strength on the adsorption ability of BBCP was quite evidencing that, as the ionic strength increases, *q*_*m*_ increases showing at the *I* = 0.5 mol L^−1^ a value more than double of that found at I → 0 mol L^−1^.
- The results of column experiments carried out with a benchtop pilot system (see Fig. 1) confirmed the highest adsorption ability of BBCP and can be considered as a first step towards the use of this adsorbent material in Oil in Water Separator systems of ships.

## Supplementary Information

Below is the link to the electronic supplementary material.

Supplementary file1 (DOCX 460 KB)

## Data Availability

The datasets used and analyzed during the current study are available from the corresponding authors on reasonable request.
