# Supplementary material for: Hydrocarbons removal from synthetic bilge water by adsorption onto biochars of dead Posidonia oceanica
Source: Environ Sci Pollut Res Int. 2022 Jul 22;29(60):90231–47. doi: 10.1007/s11356-022-21998-x (PMC9722887; doi:10.1007/s11356-022-21998-x)
Supplement: Supplementary file 1 — Supplementary file1 (DOCX 460 KB) [file 11356_2022_21998_MOESM1_ESM.docx]

Submitted to Environmental Science and Pollution Research

**Hydrocarbons removal from synthetic bilge water by adsorption onto biochars of dead *Posidonia oceanica***

Salvatore Cataldo^a+^, Nicola Muratore^a+^, Francesco Giannici^a^, David Bongiorno^b^, Vitaliano Chiodo^c^, Susanna Maisano^c^ and Alberto Pettignano^a*^

**^a^** Dipartimento di Fisica e Chimica – Emilio Segrè, Università di Palermo, V.le delle Scienze, ed. 17, 90128 Palermo, Italy

^b^ Dipartimento di Scienze e Tecnologie Biologiche, Chimiche e Farmaceutiche (STEBICEF), Università di Palermo, V.le delle Scienze, ed. 17, 90128 Palermo, Italy

^c^ Istituto CNR-ITAE, via Salita S. Lucia sopra Contesse 5, I-98126 Messina, Italy

^+^ These authors contributed equally to this work.

* Corresponding author

tel: (+39)09123897959

E-mail: [alberto.pettignano@unipa.it](mailto:alberto.pettignano@unipa.it)

**Supplementary Material**

| **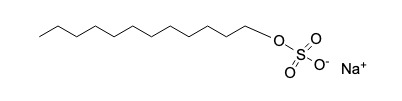** | **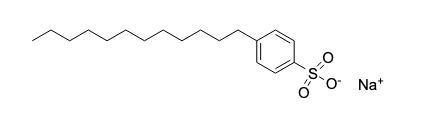** |
| --- | --- |
| SLS | SDBS |

**Figure SM1.** Molecular structure of SLS and SDBS

**Figure SM2.** TPH chromatogram of simulated bilge water after biochar (16.1 mg) treatment (top) and untreated simulated bilge water (bottom).


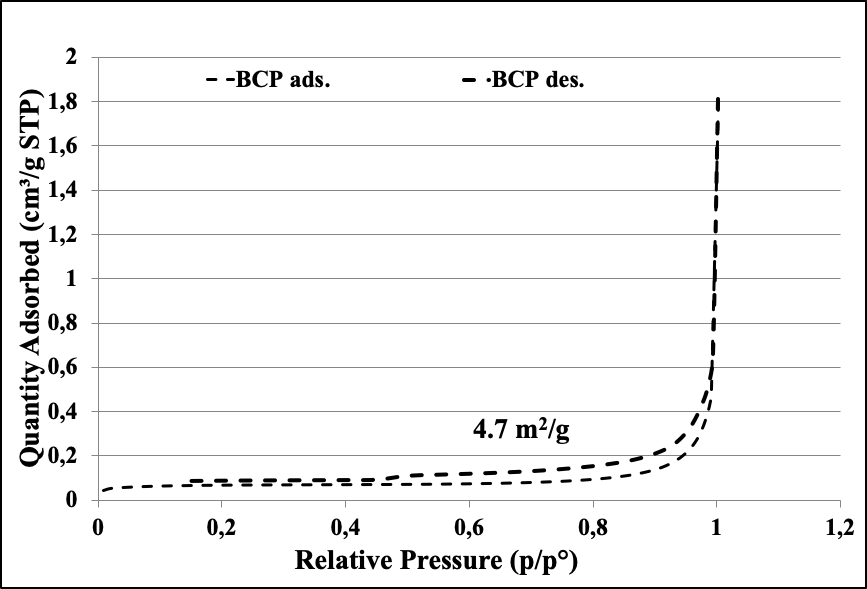

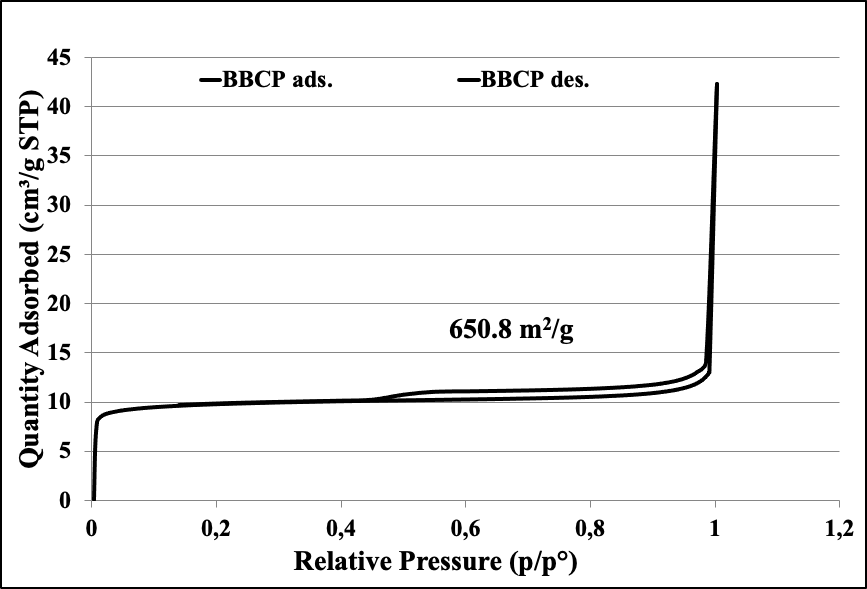


(b)

(a)

**Figure SM3.** BCP Adsorption N_2_ isotherms (a) and BBCP Adsorption N_2_ isotherms (b).


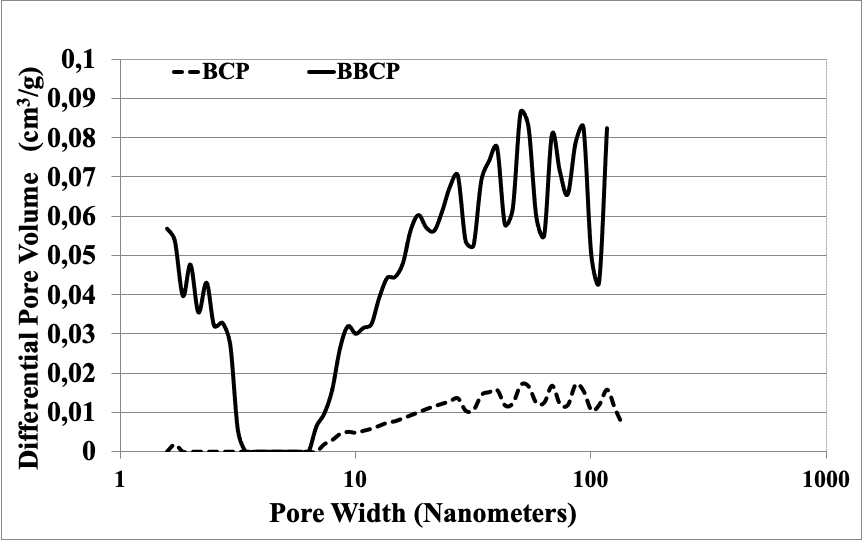


**Figure SM4.** BJH desorption pore size distribution.

**Figure SM5.** X-ray diffraction pattern of BCP. All peaks are due to the rhombohedral phase of calcite.

**Figure SM6.** Thermogravimetry differential curves of BCP (black), ABCP (red) and BBCP (blue).

**Figure SM7.** Effect of adsorbent dosage on the *q_e_* (□) and on the removal efficiency (○) of hydrocarbons for the BBCP adsorbent. Experimental conditions: 20 mL of aqueous dispersions of DMA (*c*_DMA_ ≈ 200 mg L^-1^) and SLS (*c*_SLS_ = 6 g L^-1^) (a) or SDBS (*c*_SDBS_ = 2.5 g L^-1^) (b) at pH =7 and at *T* = 25 °C.

**Figure SM8.** *q_e_* values of DMA adsorption onto BBCP at different pH values in the range 4 - 9. Experimental details: amount of BBCP = 4.4 mg; DMA emulsion: 20 mL containing *c*_DMA_≈ 200 mg L^-1^, *c*_SDBS_ = 2.5 g L^-1^, *T* = 25 °C

**Table SM1.** Calculated parameters values of Langmuir, Freundlich and Sips isotherm models for the hydrocarbons adsorption onto BCP, ABCP, FS400 and BBCP materials from dispersions containing DMA (*c*_DMA_ = 200 mg L^-1^) and SLS (*c*_SLS_ = 6 g L^-1^).

| **Adsorbent material** | **Model** | ***q_m_* (mg g^-1^)** | ***K* ^a)^** | ***n* or *s*** | **R^2^** |
| --- | --- | --- | --- | --- | --- |
| BCP | Langmuir | 11 ± 4 | 0.014 ± 0.014 |  | 0.7518 |
|  | Freundlich |  | 0.9 ± 0.9 | 2 ± 1 | 0.7669 |
|  | Sips | - | - | - | - |
| ABCP | Langmuir | 104 ± 23 | 0.014 ± 0.008 |  | 0.9364 |
|  | Freundlich |  | 9 ± 3 | 2.4 ± 0.4 | 0.9712 |
|  | Sips | - | - | - | - |
| FS400 | Langmuir | 240 ± 11 | 0.10 ± 0.02 |  | 0.9553 |
|  | Freundlich |  | 47 ± 8 | 2.9 ± 0.4 | 0.9139 |
|  | Sips | 246 ± 27 | 0.11 ± 0.03 | 1.0 ± 0.2 | 0.9515 |
| BBCP | Langmuir | 883 ± 37 | 0.15 ± 0.02 |  | 0.9814 |
|  | Freundlich |  | 174 ± 23 | 2.5 ± 0.2 | 0.9430 |
|  | Sips | 918 ± 90 | 0.16 ± 0.02 | 1.1 ± 0.1 | 0.9798 |

^a)^ K_L_ (L mg^-1^) or K_F_ (L^1/n^ g^−1^ mg^1–1/n^) or K_S_ (L^1/s^·mg^−1/s^).

**Table SM2.** Calculated parameters values of Langmuir, Freundlich and Sips isotherm models for the hydrocarbons adsorption onto BCP, ABCP, FS400 and BBCP materials from dispersions containing DMA (*c*_DMA_ = 200 mg L^-1^) and SDBS (*c*_SDBS_ = 2.5 g L^-1^).

| **Adsorbent material** | **Model** | ***q_m_* (mg g^-1^)** | ***K* ^a)^** | ***n* or *s*** | **R^2^** |
| --- | --- | --- | --- | --- | --- |
| ABCP | Langmuir | 184 ± 94 | 0.012 ± 0.021 |  | 0.5063 |
|  | Freundlich |  |  |  |  |
|  | Sips |  |  |  |  |
| FS400 | Langmuir | 309 ± 18 | 0.04 ± 0.01 |  | 0.9640 |
|  | Freundlich |  | 48 ± 12 | 3.0 ± 0.5 | 0.9480 |
|  | Sips | 338 ± 71 | 0.05 ± 0.03 | 1.2 ± 0.4 | 0.9619 |
| BBCP | Langmuir | 1106 ± 80 | 0.10 ± 0.02 |  | 0.9527 |
|  | Freundlich |  | 241 ± 47 | 2.9 ± 0.5 | 0.9286 |
|  | Sips | 1180 ± 280 | 0.11 ± 0.04 | 1.1 ± 0.4 | 0.9471 |

^a)^ K_L_ (L mg^-1^) or K_F_ (L^1/n^ g^−1^ mg^1–1/n^) or K_S_ (L^1/s^·mg^−1/s^).

**Table SM3.** Calculated parameters values of Langmuir, Freundlich and Sips isotherm models for the hydrocarbons adsorption onto BBCP from dispersions containing DMA (*c*_DMA_ = 200 mg L^-1^), SLS (*c*_SLS_ = 6 g L^-1^) and NaCl (0 ≤ *c*_NaCl_ ≤ 0.5 mol L^-1^).

| ***I* (mol L^-1^)** | **Model** | ***q_m_* (mg g^-1^)** | ***K* ^a)^** | ***n* or *s*** | **R^2^** |
| --- | --- | --- | --- | --- | --- |
| 0 | Langmuir | 883 ± 37 | 0.15 ± 0.02 |  | 0.9814 |
| 0 | Freundlich |  | 125 ± 17 | 2.0 ± 0.2 | 0.8974 |
| 0 | Sips | 940 ± 114 | 0.09 ± 0.02 | 0.9 ± 0.1 | 0.9358 |
| 0.1 | Langmuir | 1182 ± 108 | 0.05 ± 0.01 |  | 0.9523 |
| 0.1 | Freundlich |  | 122 ± 19 | 2.0 ± 0.2 | 0.9580 |
| 0.1 | Sips | 2108 ± 1259 | 0.05 ± 0.02 | 1.5 ± 0.3 | 0.9594 |
| 0.25 | Langmuir | 1489 ± 140 | 0.028 ± 0.005 |  | 0.9788 |
| 0.25 | Freundlich |  | 94 ± 5 | 1.77 ± 0.05 | 0.9965 |
| 0.25 | Sips | - | - | - | - |
| 0.5 | Langmuir | 1857 ± 279 | 0.021 ± 0.006 |  | 0.9399 |
| 0.5 | Freundlich |  | 87 ± 19 | 1.7 ± 0.1 | 0.9478 |
| 0.5 | Sips | - | - | - | - |

^a)^ *K_L_* (L mg^-1^), *K_F_* (L^1/n^ g^−1^ mg^1–1/n^) or *K_S_* (L^1/s^·mg^−1/s^).
